# Past and present marine citizen science around the globe: A cumulative inventory of initiatives and data produced

**DOI:** 10.1007/s13280-024-02119-z

**Published:** 2025-02-03

**Authors:** Uta Wehn, Ane Bilbao Erezkano, Luke Somerwill, Torsten Linders, Joan Maso, Stephen Parkinson, Christina Semasingha, Sasha Woods

**Affiliations:** 1 IHE Delft, Westvest 7, 2611 Delft, AX The Netherlands; 2 University of Amsterdam, Nieuwe Achtergracht 166, Room number: C 6.00, 1001 NH Amsterdam, The Netherlands; 3 Department of Marine Sciences, University of Gothenburg, Box 461, 405 30 Gothenburg, Sweden; 4 CREAF. Edifici C. Universitat Autònoma de Barcelona, 08193 Bellaterra, Catalonia Spain; 5 Earthwatch Europe, 256 Banbury Road, Oxford, OX2 7DE UK

**Keywords:** Marine citizen science, FAIR, Ocean, Open, Sustainability, Inventory

## Abstract

The marine environment is facing serious changes. This requires scientific understanding of the ocean’s responses to pressures and management actions to provide the foundation for sustainable development. At the same time, the production of knowledge for a more sustainable world is undergoing rapid change with the uptake of citizen science as means of opening up science. While there is increasing interest in marine citizen science, it is also often conceived as lagging behind citizen science in other areas, such as biodiversity related citizen science on land. This paper analyses empirical evidence collected >1260 past and present marine citizen science initiatives, substantiating the pervasiveness of marine citizen science around the globe. In doing so, it provides the basis for a cumulative inventory of MArine Citizen Science Initiatives (MARCSI) that can inform and shape both the growing community of marine citizen science practitioners and the science of citizen science.

**Supplementary Information:**

The online version contains supplementary material available at 10.1007/s13280-024-02119-z.

## Introduction

Serious changes such as warming, acidification, eutrophication, and loss of biodiversity are already impacting the marine environment, many of which stem from human behaviour. These changes can be projected to increase further as the human population grows towards the expected 9 billion by 2050. Scientific understanding of the ocean’s responses to these pressures and management actions can provide a foundation for sustainability transformations.

At the same time, the production of knowledge for a more sustainable world is undergoing rapid change with increasing interest in, and uptake of citizen science as a means of opening up science. Enhanced by the possibilities provided by a range of information and communication technologies for novel forms of observation and interaction, citizen science offers a unique opportunity for opening up the scientific process and for a paradigm shift in the co-creation and application of knowledge, including knowledge of marine ecosystems.

Citizen science has become increasingly popular in recent years (Kullenberg and Kasparowski 2016; Vohland et al. 2021), and has been applied in a wide range of fields. To name a few examples over the last few years alone, citizen science has been used to monitor biodiversity in protected areas (Mandeville et al. 2023), track invasive species (Larson et al. 2020), for health and biomedical research (Wiggins and Wilbanks 2019), to monitor air pollution (Mahajan et al. 2020), and even for archaeological analysis (Lambers et al. 2019).

Citizen science also encompasses a variety of forms of collaborative data and knowledge generation (Fritz et al. 2019; Haklay et al. 2021). ‘Science-driven’ initiatives are focused on scientific data and knowledge production (Bonney et al. 2009; Shirk et al. 2012); others focus on civic mobilisation and science-based citizenship (Irwin 1995) or on raising awareness, engaging the public in science and enhancing scientific literacy. Yet other citizen science initiatives aim at involving citizens and communities in monitoring activities as part of environmental management by authorities (Hager et al. 2021), sometimes alongside conservation efforts (Cooper et al. 2007; Wiggins and Crowston 2011; Schäfer and Kieslinger 2016). Community-based initiatives are led and undertaken by community members with collaboration and support of experts or scientists (Whitelaw et al. 2003; Danielsen et al. 2022). Data collection is considered a core activity of any form of citizen science (Haklay et al. 2021). The increasingly multi-stakeholder, multi-dimensional nature of citizen science initiatives (Hager et al. 2021; Wehn 2022) is paralleled by discussions (that we do not rehearse here) on a broader, more inclusive and holistic understanding of what is (and what is not) citizen science and the realisation that citizen science needs to be characterised context (Haklay et al. 2021).

Another area of change within marine science is the increased digitisation and focus on modelling ocean processes. This has been epitomised by the growing interest in digital twins of the ocean (DTOs), with programmes exploring the development of DTOs from both the United Nations (Bahurel et al. 2023) and European Union (Bye et al. 2022). A digital twin is a virtual replica of a real-world object, system, or process (in this case, the ocean). Extensively used in engineering and manufacturing, digital twins are increasingly being used to capture natural world properties and behaviours, providing a safe and controlled environment to simulate scenarios, monitor and analyse impacts on oceans. Fed by continuous real-time and historical observations from thousands of sensors across the world’s oceans and numerous satellites, at the same time allowing for integration of alternative data flows, the Digital Twin of the Ocean should integrate advanced modelling powered by artificial intelligence and machine learning and supercomputing. Currently, two EU-funded projects are currently creating digital twins of the ocean: EDITO (2022) and ILIAD (2022). Whilst they have potential to support decision makers in advancing ocean management, DTOs still face implementation barriers around data availability, quality, and compatibility (Tzachor et al. 2023). Citizen science has been proposed as a potential data source to support DTOs but the potential of citizen science in this context is yet to be fully realised (Bye et al. 2023). A key requirement for integrating citizen science initiatives into DTOs or other modelling infrastructures is that they implement data management documentation that reflects on how to make data and initiatives more scientifically sound, improve data quality and openness, and increase reproducibility (Thuermer et al. 2023) by implementing findable, accessible, interoperable and reusable (FAIR) principles (Wilkinson et al. 2016). While the FAIR principles have become popular, the way to demonstrate conformance to them is not always well understood resulting in many data initiatives claiming to be FAIR without any testing. A strict interpretation of the go-fair sub-principles indicates that the “F” (findable) is demonstrated by comprehensive metadata and the existence of persistent identifiers, the “I” (interoperable) is demonstrated by the use of data vocabularies and agreed semantics, and the “R” (reusable) is made possible by the selections of a clear and open licence (Thuermer et al. 2023). One of the few examples of an actual methodology to assess the FAIRness of a citizen science initiative is the Kakila database which contains observations of whales in the Guagalupe archipelago. In their paper, they present a detailed list of actions implemented to comply with each of the four FAIR principles (Coché et al. 2021).

Marine science cuts across a range of disciplines and sectors. As such, it provides particular complexity, challenges and opportunities for opening up. The analysis presented in this paper builds on a series of previous scientific articles and reviews that contain inventories of marine citizen science initiatives. Such reviews display a wide variety of approaches in terms of goals, methodological orientation, analytical depth, and geographical scope. Although most review studies share a specific focus on marine citizen science, their scope is often limited to particular regions, such as the North Sea (van Hee et al. 2020), the Baltic Sea (de Grunt 2021), the Mediterranean Sea (Giovos et al. 2019) and Europe (Garcia-Soto et al. 2021). In general, most studies tend to focus on the Western world and developed countries (e.g., Earp and Liconti 2020; Kelly et al. 2020). As well as geographic limitations, many of the previous reviews have been restricted to a specific thematic focus including marine litter (Popa et al. 2022; Kawabe et al. 2022), biodiversity (Theobald et al. 2015), and coastal initiatives which include remote public engagement (Lucrezi 2021).

Against this background, the collective contribution by Thiel et al. (2014)—which identified 227 marine citizen science studies before 2014—and Sandahl and Tøttrup (2020)—which identified a further 185 studies published between 2014 and 2018—remains an isolated (yet already outdated) attempt to provide a comprehensive overview of marine citizen science at the global level. In our paper, we build upon this work by providing a state-of-the-art inventory of marine citizen science initiatives worldwide. We go beyond previous reviews in terms of geographical scope, temporal scale, and topical breadth included, identifying and analysing 1267 marine citizen science initiatives. As such, we believe that our contribution can address calls for the development of a “network of marine citizen science projects globally, to improve the exchange of relevant support and information and enhance capacity, particularly in underrepresented regions, including Asia and Africa” (Kelly et al. 2020).

The main aim of this paper is to provide comprehensive empirical evidence of the pervasiveness of marine citizen science, which is often assumed to be underrepresented compared with citizen science in terrestrial or freshwater environments (Theobald et al. 2015). Furthermore, the efforts resulting from this paper have created the basis for a cumulative and dynamic online inventory of marine citizen science initiatives (MARCSI), allowing these initiatives (and their data) to be easily findable, and further connecting the marine citizen science community.

In this paper, we present the findings of a detailed review of marine citizen science initiatives globally. Firstly, we detail the methodological process behind the development of the global marine citizen science inventory in terms of its structure as well as the literature review and other search efforts undertaken to identify relevant marine citizen science initiatives. We then highlight the results of the analysis of the identified marine citizen science initiatives and the ‘FAIRness’ of their data. The implications of these results are then discussed, and finally concrete conclusions are identified.

## Materials and methods

### Marine citizen science inventory structure

The structure of the marine citizen science inventory was created by combining relevant characteristics and concepts from recent citizen science inquiry efforts as well as marine citizen science reviews. From the field of citizen science, defining elements were identified from the *Data Standard for Public Participation in Scientific Research (PPSR)* (Shirk et al. 2012) (see Table S1 for the detailed structure).

**Fig. 1:**
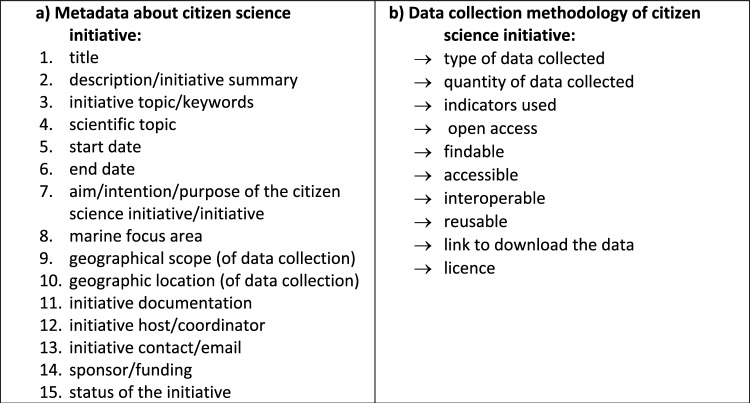
List of fields in the MARCSI inventory

The metadata section of the inventory consists of fifteen fields, illustrated in Figure 1a, for each marine citizen science initiative. Twelve of these fifteen fields have a correspondence with the PPSR Project Metadata Model (PMM) metadata fields. Three fields, namely the scientific topic addressed by the initiative, the specific marine focus area and the funding organisation are additional compared to the PPSR metadata. The ‘data collection methodology’ section of the inventory is illustrated in Figure 1b.

Initially, there was the intention to also capture information related to impact using the fields of the inquiry instrument of the Citizen Science Impact StoryTelling Approach (Wehn et al. 2021). The method requires capturing several fields describing the problem that the initiative tries to solve, the governance dimensions, challenges and wider impacts. The population of these fields was not possible from the available written initiative descriptions, and would require a more thorough investigation that was considered impractical due to the number of initiatives to assess.

The scientific topics outlined in Garcia-Soto et al. (2021) consist of the following categories: *archaeology* (e.g. maritime history), *biodiversity* (e.g. collecting information on many different species, for instance, to map the biodiversity of a specific region), *ecology* (e.g. coastal ecology, the state of certain habitats, species interaction with the habitat, or the impact of climate change on the ecosystem), *environmental variables* (e.g. water quality, temperature or sea level rise), *fisheries* (e.g. fishery catches or fish stocks), *pollution* (e.g. marine litter or the effect of oil spills on birds.), *single* species (e.g. marine mammals, fish, birds, seaweeds, plankton, molluscs, crustaceans, invasive species), *not specified*, and *other*.

The marine focus area includes eight categories: on shore; near shore; offshore; on shore & near shore; on shore & offshore; near shore & offshore; on shore & near shore & offshore; don't know/not specified. Onshore is defined as land above the high tide line. Nearshore is defined as the intertidal zone plus the area out to 1 nautical (1856 m) outside of the ‘baseline’. The baseline is a concept used in maritime law, including in UNCLOS (1982). Primarily, it is the low tide line (‘normal baseline’). A country can also define ‘straight baselines’ between outermost islets and grounds. Many countries make extensive use of straight baselines around their archipelagos. Offshore is defined as the area further outside of the baseline than 1 nautical mile. The above definition of the limit between nearshore and offshore was chosen because 1) it coincides with the limit of the EU Water Framework Directive, and 2) one nautical mile is approximately how far one can observe the sea (depending on tools and objects of interest).

Moreover, the geographical scope was divided into five categories: *global*—multiple focus areas, anywhere in the world; *international*—focus area is in multiple, specific countries (e.g. the Netherlands, Belgium, France), *national*—focus area is spread across one country (e.g. the Netherlands), *regional*—focus area is spread across a large area within one country (e.g. a US state), *local*—focus area is specific to a small location within one country (e.g. a city or beach).

### Search for marine citizen science initiatives

The search for past and present marine citizen science initiatives consisted of a strategic, multi-source effort that maximised the efficiency of limited available resources that could be used to ascertain that a given marine citizen science initiative exists or had existed in the past. This strategy resulted in efforts that spanned (a) literature searches, (b) citizen science-related organisations and platforms, (c) events (conferences and workshops) and (d) social media (namely, X (previously Twitter)). We explicitly excluded other search strategies such as surveys among marine citizen science coordinators, in order to be able to ascertain the extent to which identified initiatives practice the FAIR principles, without relying on personal contact with coordinators.

Citizen science-related organisations, marine and oceanographic organisations, and main citizen science platforms were searched from July 2021 till spring 2024 (see Table 1). Among these, the online^1^ (a joint initiative by three EU-funded projects) was the most prominent, resulting in 304 initiatives identified as potentially for our study.

**Table 1:** Sources of information about past & present marine citizen science initiatives

Cluster	Title of source	Webpage
Citizen science related organisations	Australian Citizen Science Association (ACSA)	https://biocollect.ala.org.au/
	Citizen Science Association (CSA)	https://citizenscience.org/
	CitizenScience.gov	https://www.citizenscience.gov/
	Earthwatch	https://earthwatch.org.uk/
	European Citizen Science Association (ECSA)	https://ecsa.citizen-science.net/
	GEOSS Portal	https://www.geoportal.org/
	OPEN (Observatories Participatifs des Especes et de la Natura)	https://www.open-sciences-participatives.org/
Marine and oceanographic organisations & platforms	Australian Coastal Research	https://coastalresearch.csiro.au/
	National Centre coastal ocean science (NCCOS)	https://coastalscience.noaa.gov/
	National Oceanic and Atmospheric Administration (NOAA)	https://www.noaa.gov/
	Ocean Sanctuaries	https://oceansanctuaries.org/
	Reef Environmental Education Foundation (REEF)	https://www.reef.org/
	Marine Biological Association	https://www.mba.ac.uk/
	Marine Conservation Society	https://www.mcitizenscienceuk.org/
	Sea Grant and Community Science	https://seagrantcommunityscience.msi.ucsb.edu/
	Wavelinks	https://wavelinks.inventory
Main citizen science platforms	EU-citizen.science	https://eu-citizen.science/
	iNaturalist	https://www.inaturalist.org/
	MICS platform	https://mics.tools/
	Project Noah (Citizen Science Platform)	https://www.projectnoah.org/
	SciStarter	https://scistarter.org/
	Zooniverse	https://www.zooniverse.org/

Relevant literature was identified based on the method outlined by Moher et al. (2009). The search for scientific papers was done in Google Scholar, Scopus and Web of Science. Keywords were referred to two distinct aspects of the literature: (1) different terms used to refer to citizen science and (2) the field of marine science (see Table 2). Based on that, a set of keywords that refer to both aspects were identified. The Boolean operator AND was used to combine different terms and the asterisk character (*) was added to ensure the inclusion of variations on each of the terms (see Table 2).

**Table 2:** Parameters used in the literature search

Field of citizen science	Field of marine science
Citizen science, public engagement, stakeholder engagement, citizen observator*, community science, community-based monitoring, crowd science, crowd*, eparticipa*, participatory action research, participatory monitoring, participatory sensing, public participa*, people-centric sens*, Volunteered geographic information, data	Ocean, marine

**Fig. 2:**
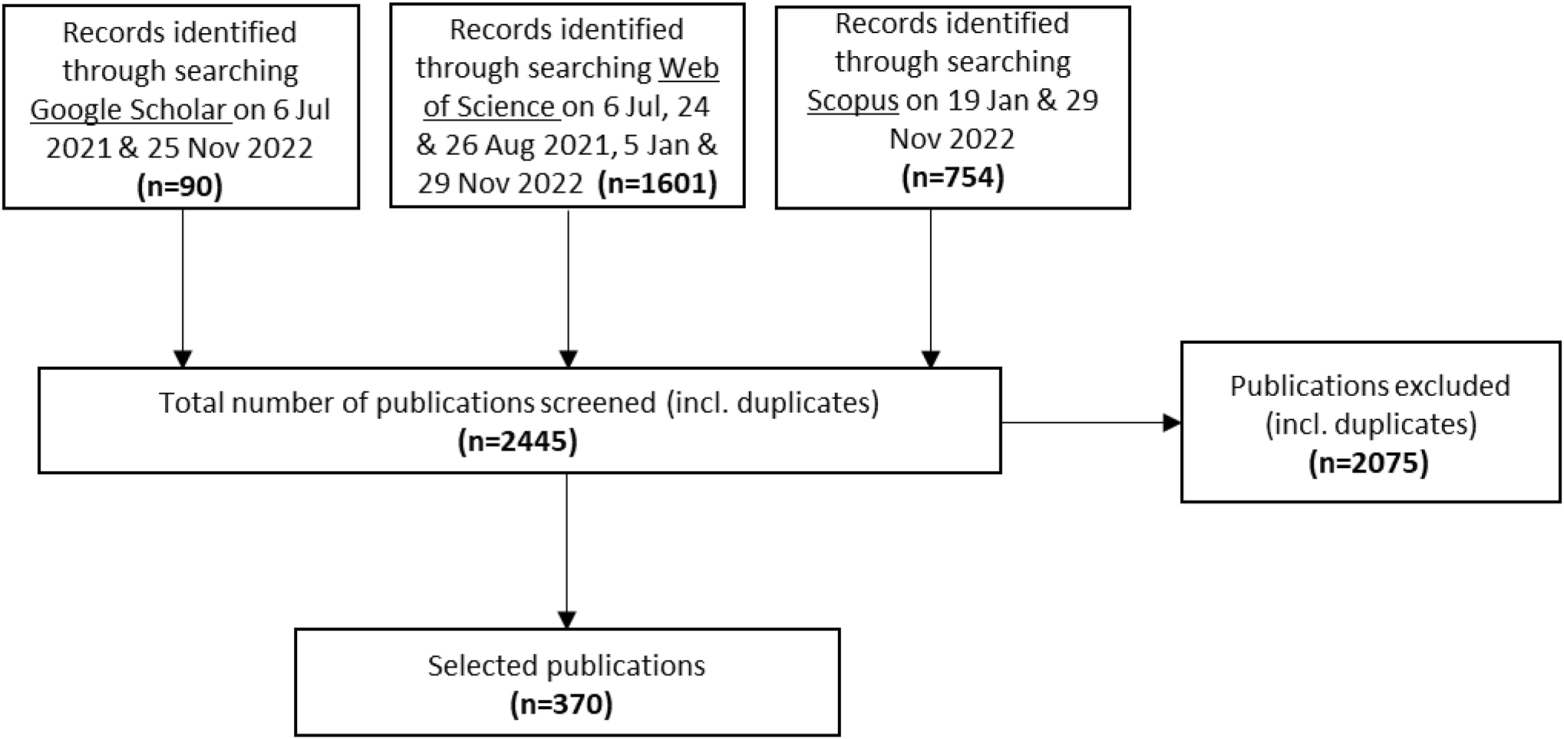
Summary of the steps for selecting relevant publications

Overall, the search on Google Scholar returned 90 records, 1601 in the Web of Science and the process in Scopus 754 records. In total, the literature search resulted in 2445 records (See Fig. 2). After removing duplicates and screening for relevance in the title, abstract and full-text review in terms of the broad definition of citizen science applied in this study (see introduction), 370 publications were selected as relevant publications when they reported about one or more marine citizen science initiative and were included in a Mendeley Library. The eligibility criteria for inclusion in the final list was in line with the purpose of the analysis, i.e. to create an inventory on marine citizen science initiatives, past and present. Articles that were found to be unrelated to marine citizen science (despite the initial screening), using the broad definition of citizen science initiatives (see introduction), were excluded from the further analysis.

From the analysed publications, several publications were considered key references for identifying multiple relevant marine citizen science initiatives, programmes and initiatives, since they contained *reviews* of marine citizen science initiatives (at different scales): Thiel et al. (2014), Theobald et al. (2015), Garcia-Soto et al. (2017), Giovos et al. (2019), Earp and Liconti (2020), Kelly et al. (2020), Sandahl and Tøttrup (2020), van Hee et al. (2020), de Grunt (2021), Garcia-Soto et al. (2021), Lucrezi (2021), Kawabe et al. (2022), and Popa et al. (2022).

Additional initiatives were identified from conferences (e.g. AltantOS Ocean Hour conference) and workshops (e.g. Cos4cloud Workshop 'Engaging the society beyond data collection’). Also, in May 2022, a Twitter account was created (@Citsci_marine) to engage with the marine and citizen science community in order to identify marine citizen science initiatives. This resulted in the identification of 2 new initiatives, while confirming the existence of 10 initiatives already included in the inventory.

In total, 1403 potential marine citizen science initiatives were identified from July 2021 to March 2024: 676 marine citizen science initiatives were identified via publications (original research articles and reviews) (48.2%), 716 from platforms (51%) and 11 via opportunistic identification (0.8%).

### Compilation of information about marine citizen science initiatives

The identified cases were added to the inventory and categorised into one or more relevant scientific topics, based on the data collected and/or the description of the initiative. The general information was completed based on the information obtained, e.g. if an initiative was mentioned or described in a scientific publication, it was complemented by information found on the initiative’s own website. Five of the authors added information to the inventory and flagged potential duplicates, cases that could not easily be categorised as relevant for the purpose of our study or initiatives for which no detailed information could be found (75 cases). Finally, all authors discussed the flagged cases, removed duplicates from the inventory and ensured consistency in the application of the criteria. In total, 82 cases were discussed, of which 21 remained in the inventory and 61 were removed, erring on the side of caution. This resulted in the data set of 1267 cases. The data set created, i.e. the inventory of marine citizen science initiatives, is available on Zenodo (Wehn et al. 2024).

In the context of this study, focusing on marine citizen science initiatives to assess the pervasiveness of marine citizen science globally, we included initiatives regardless of their leadership, i.e. whether they were science-driven, community-driven, NGO- or authority-initiated, or their purpose, e.g. contribution to science, educational purposes or conservation. We focused on initiatives that engage volunteers in data collection (or further steps of the scientific method) over time, and excluded one off events such as so-called blitzes or hackathons.

The Topic Category entries done by two co-authors from environmental sciences were scrutinised additionally by a marine scientist, indicating their level of (dis)agreement with the categorisation as well as their level of confidence in their own (alternative) assessment. A subset of 150 (15%) initiatives was created by randomly selecting initiatives from the entire inventory dataset. This subset was assessed in detail by the marine scientist based on the four fields ‘Type of data collected’, ‘Data collection methodology’, ‘Description/Initiative summary’ and ‘Initiative topic/keywords’. In 23 (16%) of the 150 double-checked initiatives, the marine scientist disagreed with the (primary) classification done by the generalists, *however, mostly with low to moderate confidence in their own assessment*. Only for 7 (5%) of the 150 initiatives, did the marine scientist disagree with high confidence with the assessment of the environmental scientists. Based on this assessment, it was concluded that the entire dataset did not need to be double-checked by the marine scientist, given that the level of disagreement was relatively low and the time taken to review all initiatives by the marine scientist was very high. It was therefore decided that no changes would be made to the topic classifications in the dataset. A fourth author cross-checked the entries for the start and end dates of all projects in spring 2024 and updated these if needed.

The Marine focus area (onshore/nearshore/offshore) entries were likewise additionally scrutinised by a marine scientist. The principles seem straightforward and meaningful. However, the marine scientist nevertheless often found categorisation of the marine focus areas difficult. In a tested subset of 10 % of the projects, the marine scientist made different categorisations from the original ones made by the environmental scientists in 38% of the subset. Both when the marine scientist agreed and disagreed, it was often with only moderate confidence. Often the disagreement related to a multi-area categorisation, e.g. near-shore versus near-shore & offshore.

The focus of this study is the marine domain, which we delimit by only including ‘marine’ and ‘ocean’ in the search for fields of marine science, but not ‘coastal’, see Table 2. We nevertheless define an ‘onshore’ focus area among the initiatives in the inventory. Including ‘coastal’ as a field of marine science would have multiplied the number of hits, most of which we would then have to manually exclude as non-marine. Furthermore, our study is using the initiatives’ own definitions and descriptions of their initiatives. An initiative that describes itself as coastal without mentioning ‘marine’ or ‘ocean’ must hence be treated as non-marine.

In many cases the project descriptions do not reveal a clear focus area, or it requires significant understanding about the project to categorise the focus area. Our conclusion is that the focus area of marine citizen science projects is difficult to categorise with high confidence. Reaching high confidence in categorisation would require in depth investigations beyond the scope of the current study. Neither is it obvious that project leaders/participants would define their marine focus areas according to the principles used herein. A case in point are projects dealing with seabirds or litter (as mentioned above), where all data collection may be onshore but the scientific focus may very well be to understand issues far offshore. This ambiguity of a project focus area is of course not a specific feature of citizen science.

### Data analysis approach

The key data about each Marine Citizen Science initiative in the inventory (namely Initiative Start Year, Topic Category, Marine Focus Area and Coordinating Organisation) were extracted into a Microsoft Excel document. For Initiative Start Year, Topic Category and Marine Focus Area, the number of occurrences of each were counted, in order to identify the frequency of each. The categorisation of the length of the initiatives was calculated using the Initiative Start Year and Initiative End Year entries. For the ‘Coordinating Organisation’ data, each identified organisation was categorised into one of the following: Research organisation (incl. universities); NGO; Private Sector Organisation; Government Institute; International Organisation; or Other. These categories were then counted for frequency. All graphs were created using Microsoft Excel.

## Results

**Fig. 3:**
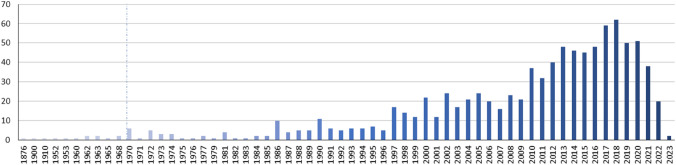
New marine citizen science initiatives initiated per year (1876–2023) (*n* = 937)

In total, the existence of 1267 (past or present) marine citizen science initiatives could be ascertained and detailed descriptive information completed. As reported in previous studies (e.g. Garcia-Soto et al. 2021), the findings of this study emphasise the significant increase in the popularity of marine citizen science in the late 1990s and into the 2000s (see Fig. 3).

**Fig. 4:**
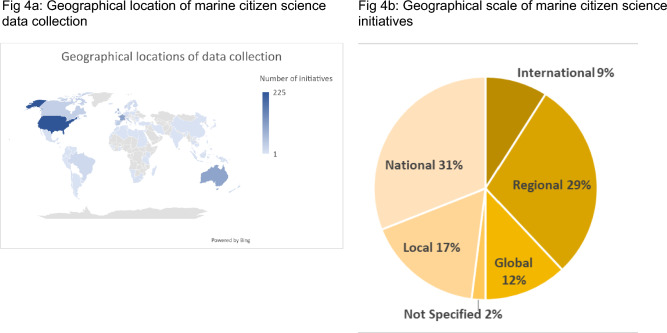
Geographical placement of marine citizen science initiatives (*n* = 1267) **a** Geographical location of marine citizen science data collection **b** Geographical scale of marine citizen science initiatives

The spread of the identified initiatives around the globe is evident (Fig. 4a), with data collection taking place at diverse scales, ranging from local (17%), national (31%), regional (29%), international (9%) to global (12%) (Fig. 4b).

**Fig. 5:**
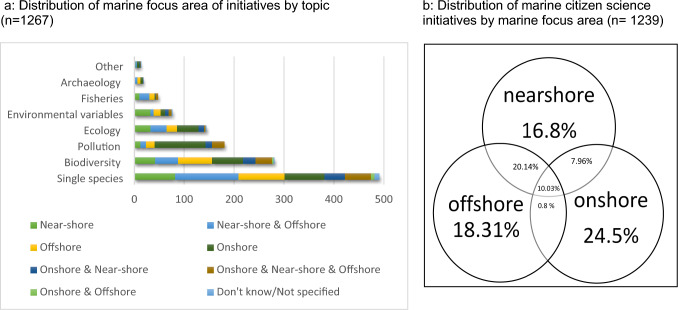
Marine citizen science initiatives by marine focus area **a** Distribution of marine focus area of initiatives by topic (*n* = 1267) **b** Distribution of marine citizen science initiatives by marine focus area (*n* = 1239)

A large proportion of the initiatives identified focused on issues concerning single species (39%) (see Fig. 5a). The majority of the rest of the initiatives focused on matters of biodiversity (22%), pollution (14%) and ecology (11%), with relatively few focusing on environmental variables (6%), fisheries (4%), archaeology (1%) or other topics (1%). While these results are mostly consistent with those of Garcia-Soto et al. (2021), proportionally fewer ‘single species’ initiatives were identified in this review, leading to a slightly more even spread of topics. The marine focus areas of these initiatives (see Fig. 5b) are split between ‘on shore’ (24.5%), ‘near shore and off shore’ (20.1%), ‘near shore’ (16.8%) and ‘off shore’ (18.3%). Relatively few initiatives focus on all three focus areas (10%) or on ‘on shore and near shore’ areas (almost 8%). No initiatives were found that focus on only ‘on shore AND off shore’ areas. The interactions between the topic category and marine focus area were also investigated (see Fig. 5a). One notable trend that was identified was that ‘on shore’ initiatives comprise over half of all pollution-focused citizen science initiatives, largely due to the various beach clean initiatives organised worldwide.

The duration of the initiatives could be calculated for the majority of the identified marine citizen initiatives (67%; 859) (see Table 3). From those, 507 were still ongoing at the time of writing (2024). About 7% (90) were categorised as lasting less than one year, of which 24 were one-off initiatives (single surveys or blitzes), and the rest were active for more than one month (but less than one year). One third of those initiatives (271) last(ed) between 1 and 5 years (including those still ongoing at the time of writing), a typical life-time of project-funded citizen science initiatives. More than half (466) last(ed) longer than 5 years, of which 403 are still ongoing. More than 50 initiatives (54) have a duration of more than 30 years (listed in Table 4), with ‘The Conchological Society of Great Britain and Ireland Marine Mollsuc Recording Scheme’ being the longest, running since 1876 (147 years). Together, the findings regarding the duration of the identified initiatives confirm the findings of Thiel et al. (2014) and serve to dispel the myth—at least for marine citizen science—that most citizen science initiatives are short lived.

**Table 3:** Duration of marine citizen science initiatives (n=859) For the 403 other initiatives, no start and/or end date were found

Duration range (years)	# of marine citizen science initiatives	# of which are ongoing (in 2024)
<1	90	N/A
1–5	271	104
6–10	186	145
11–15	105	95
16–30	121	114
Above 31	54	49
Total	859	507

**Table 4:** Overview of identified marine citizen science initiatives with duration >30 years (n = 54)

Marine citizen science initiative title	Start date	End date	Initiative duration (years)
The Conchological Society of Great Britain and Ireland Marine Mollsuc Recording Scheme	1876	Ongoing	147
Audubon Christmas Bird Count	1900	Ongoing	123
Marine oil pollution and beached bird surveys	1910	2000	90
Recreational spearfishers for collecting marine data in south-eastern Australia	1960	Ongoing	63
Cooperative Shark Tagging Program	1962	Ongoing	61
Cooperative Billfish Tagging Program (CBTP)	1963	Ongoing	60
American Littoral Society Fish Tagging Program	1965	Ongoing	58
Suivi des oiseaux échoués sud Baie de Somme	1970	Ongoing	53
Enquête oiseaux échoués Picardie	1970	Ongoing	53
Réseau National Échouages (RNE)	1970	Ongoing	53
Alaska Groundfish Tagging Map	1972	Ongoing	51
Project Oceanology	1972	Ongoing	51
Royal Society for the Protection of Birds Beached Bird Survey	1972	Ongoing	51
Waterbirds in Western Port	1973	Ongoing	50
International Shorebird Survey	1974	Ongoing	49
Atlantic Canada Shorebird Survey	1974	Ongoing	49
South Carolina Marine Game Fish Tagging Program (MGFTP)	1974	Ongoing	49
Victorian Wader Study Group	1975	Ongoing	48
Porcupine Marine Natural History Society Recording Scheme	1976	Ongoing	47
Southeastern US Marine Mammal Stranding Network	1977	Ongoing	46
Cape Clear Bird Observatory	1971	2017	46
A long way from home: Biosecurity lessons learnt from the impact of La Nina on the transportation and establishment of tropical portunid species	1970	2014	44
Aquatic Mammal Rescue Program, Brazil	1981	Ongoing	42
Australasian Wader Studies Group (AWSG)	1981	Ongoing	42
West Coast Marine Mammal Stranding Network	1981	Ongoing	42
US National Park Service Kelp Forest Monitoring Program	1982	Ongoing	41
Blue Fish Tagging Study	1963	2002	39
Alliance for the Chesapeake Bay (Rivertrends)	1985	Ongoing	38
Solitary Islands Underwater research Group Inc. (SUrG)	1985	Ongoing	38
Coalition for Buzzards Bay - Baywatchers	1986	Ongoing	37
Programme d'études et de protection des phoques en baie de Somme	1986	Ongoing	37
Mussel Watch	1986	Ongoing	37
International Coastal Cleanup by Ocean Conservancy	1986	Ongoing	37
Coral Cover in the Indo-Pacific	1968	2004	36
Coastwatch Ireland Coastal Survey	1987	Ongoing	36
Coastwatch Europe	1987	Ongoing	36
Sapphire Coast Marine Society Surveys	1988	Ongoing	35
Seasearch	1988	Ongoing	35
Beach Sweep	1988	Ongoing	35
Mississippi Coastal Cleanup	1988	Ongoing	35
LagoonWatch Monitor	1989	Ongoing	34
The Stranding Network of Schleswig-Holstein	1989	Ongoing	34
Friends of Casco Bay	1989	Ongoing	34
The Delaware Bay Horseshoe Crab Survey	1990	Ongoing	33
Great Bay Coast Watch	1990	Ongoing	33
Billfish Foundation Tagging	1990	Ongoing	33
UK Cetacean Strandings Investigation Program	1990	Ongoing	33
Marine animals research & intervention network (MARIN)	1990	Ongoing	33
Tag/Flag Tournament	1990	Ongoing	33
Sea Watch Foundation—report a sighting	1991	Ongoing	32
Surveillance estivale des Phoques veaux-marins	1991	Ongoing	32
Strawberry Isle Marine Research Society—Killer Whale Monitoring	1991	Ongoing	32
The University of Delaware Citizen Monitoring Program	1991	Ongoing	32
Queensland Wader Study Group	1992	Ongoing	31

**Fig. 6:**
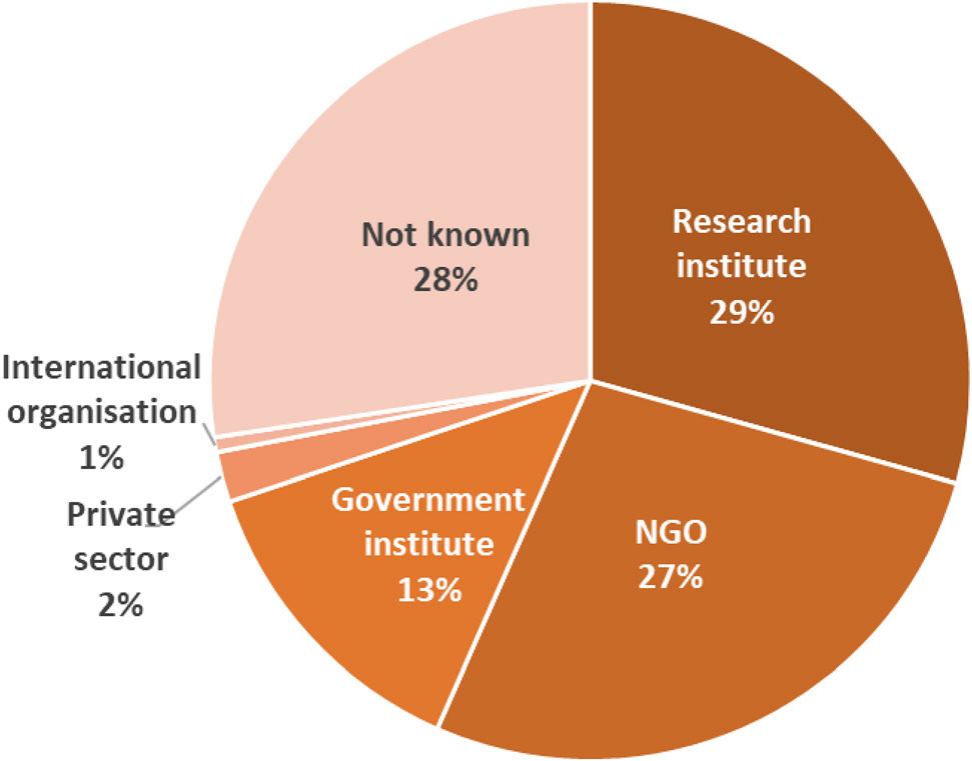
Distribution of marine citizen science initiatives per type of lead organisation (*n* = 1267)

This review found that, for those initiatives for which the coordinators could be identified, Research organisations (29%) and NGOs (27%) coordinate the majority of the identified marine citizen science initiatives, while government institutes coordinate 13% of these initiatives (see Fig. 6). In comparison, private sector organisations (2%), international organisations (1%), and other types of entities (<1%) coordinate relatively few initiatives.

**Fig. 7:**
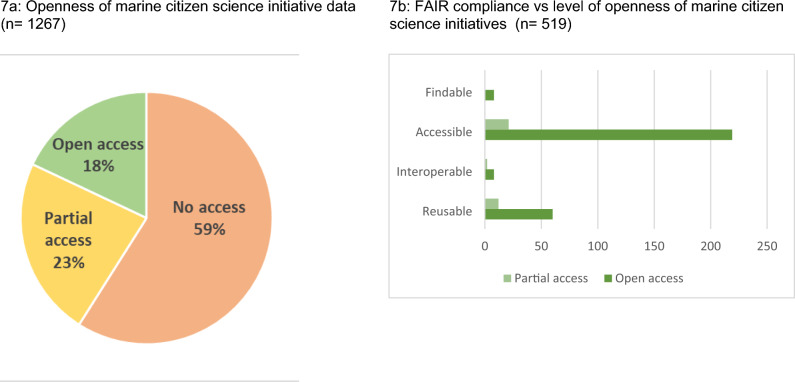
FAIR compliance and level of openness of marine citizen science initiatives, **a** Openness of marine citizen science initiative data (*n* = 1267) **b** FAIR compliance vs level of openness of marine citizen science initiatives (*n* = 519)

Finally, the openness of the data produced by the initiatives was assessed (see Fig. 7a). Although it is possible to comply with FAIR without offering data openly, opaque datasets do not allow us to test the extent of compliance with the FAIR principles with the restrictive interpretation posed in the introduction, so the FAIR analysis was applied only to the 535 initiatives that are not closed (defined as initiatives that neither provide access to the raw data (for example in a CSV file) nor partial access to the data (e.g. via a PDF in a report). The analysis found that 18% of the initiatives claim to provide full open access to the raw data collected, 23% gave partial access (for example, via a report, paper or factsheet), while 59% of initiatives gave no access to their data at all.

In only 2 marine citizen science initiatives we were able to demonstrate compliance with all 4 aspects of the FAIR principles according to the restrictive interpretation. Furthermore, if we apply a strict criteria for the “I” principle and we require that initiatives provide a clear link to standard vocabularies to define the observed properties, only 10 initiatives (1.8%) can be considered fully semantically interoperable; it is even possible that many more use common concepts that allow for data aggregation with other initiatives without providing a reference to a standardised vocabulary. Standardised vocabularies (ontologies) are explicit and formal specification of mental concept abstractions that allows alignment, mapping and relationships between concepts such as "same As", "narrower Than", and "broader Than" (Bermudez et al. 2005). To increase interoperability, projects should use pre-existing ontologies (such as the ones Marine Metadata Interoperability) and prevent new vocabulary proliferation. Regarding “reproducibility”, many initiatives do not provide a reference to a licence associated with the data. The lack of reference to a licence creates a barrier to data reusability; without a licence, a possible user does not have any clear way to know what is authorised to do with the data. Indeed, only 73 projects provide a well described licence: 20 adopting the Creative Commons (CC0), 19 require attribution (CC-BY), 7 does not allow commercial activities (CC-NC), and 4 require contacting the producer before using the data. Less than 46% of non-closed initiatives provide an easy mechanism to access their data and only 2.4% provide a persistent identifier (a DOI—digital object identifier) for making data findable for humans and computers (see Fig. 6b).

## Discussion

The findings of this study present a detailed picture of the current state of marine citizen science globally. With more than 1260 initiatives that can be described in detail and defined as marine citizen science, this study demonstrates the substantial size and growth of marine citizen science initiatives around the world. In this respect, it serves to counter doubts regarding the relevance of marine citizen science initiatives in their own right (e.g. Theobald et al. 2015) and in comparison to other thematic areas of citizen science on land (including those linked to freshwater resources), presenting instead a ‘ship of opportunity’ in the marine observation field.

Thematically, this study reveals that marine citizen science initiatives are largely focused on individual species, while many initiatives are also working on issues of biodiversity and pollution. Relatively few initiatives focus on other topics (such as environmental variables, fisheries or archaeology), despite some examples showing promising results for citizen science in these areas. These results largely support the findings from previous studies in the field (e.g. van Hee et al. 2020). In the present study, the scientific topic categorization was first done by environmental scientists, and then assessed once more by a marine scientist based on entries in four fields of the inventory. In general, this double assessment revealed relatively little disagreement between the environmental scientists and the marine scientist. What the detailed check of the topic categorization did reveal was the inherent ambiguity and subjectivity allocating one or more scientific topics to a given marine citizen science initiative. This is not surprising, given that the categories are at the same time overlapping, non-exhaustive and not strictly defined in academic literature and research. For example, the category ‘ecology’ can arguably include most of the initiatives. Another very frequent difficulty is to categorise marine citizen science initiatives that focus on more than one species, whereby neither ‘single species’ nor ‘biodiversity’ may be suitable: merely observing more than one species does not qualify as what is typically understood as observation of biodiversity. We conclude that this difficulty may have applied to the previous reviews of marine citizen science initiatives although this was not explicitly discussed in those papers (van Hee et al. 2020; Garcia-Soto et al. 2021). The categorisation provided by Thiel et al. (2014) provides an alternative thematic stratification (fauna, flora, contamination, oceanography, geology) but it is questionable whether this would serve better to allocate marine citizen science initiatives to specific thematic topics.

When examining the distribution of initiatives by type of coordinating organisation, the results of this diverge from the findings of previous studies. This study found that research organisations (including universities) and government organisations coordinate more, and NGOs far fewer (while still being the largest group) than suggested by Thiel et al. (2014) and van Hee et al. (2020). The role of the private sector was also identified (albeit to a minor extent), a group not identified by Thiel et al. (2014) and van Hee et al. (2020).

Previous reviews have found a high proportion of marine citizen science initiatives monitoring “on shore” environments. The reported proportion of “on shore” initiatives ranges from 34 (Earp and Liconti 2020) to 60% (van Hee et al. 2020). It has been suggested that this pattern is due to the relative accessibility of the ‘on shore’ environment for citizen scientists compared to ‘off shore’ areas (Garcia-Soto et al. 2021). This marine area bias is especially prevalent within particular topic categories such as pollution; a review of marine litter initiatives found that over 70% of initiatives involved data collection on shore (Kawabe et al. 2022). To a certain extent, these findings have been confirmed by this review, with a high proportion of initiatives taking place ‘on shore’ (41%) and a notable number of ‘on shore', pollution-focused initiatives (largely due to the popularity of beach clean-up initiatives). However, in contrast to previous reviews, initiatives were categorised as working across multiple areas (for example, monitoring both ‘on shore’ AND ‘near shore’ environments). This makes it harder to compare to previous findings as, for instance, only 26% of initiatives were found exclusively in ‘on shore’ areas which is significantly lower than previous reviews. Similarly, almost half of initiatives (48%) were found to involve at least some ‘off shore’ element which is significantly higher than previous reviews which found <10% of initiatives ‘off shore’ (Thiel et al. 2014; Earp and Liconti 2020). The results of this review therefore suggest a more even distribution of initiatives across marine areas than previous reviews and challenges the assumption that most marine citizen science initiatives occur only “on shore”. For completeness, every combination of ‘on shore’, ‘near shore’ and ‘off shore’ was considered as a category in this review, including the option ‘on shore and off shore’ (not including near shore). In practice, this combination of marine areas is very unlikely. Indeed, no initiatives fitting this category were found during this review. Future reviews might therefore consider omitting this category unless exceptions can be found.

Overall, the efforts undertaken by this study illustrate that the step from knowing a marine citizen science initiative exists to accessing its data is still considerable. With respect to how open and FAIR the data of marine citizen science initiatives are, this study identified a large percentage of marine citizen science initiatives with ‘non-open’ data, with only 20% of initiatives making their data fully open and 23% partially publishing data. With a combined 43%, this number is significantly lower than the average for citizen science initiatives at large, with a recent study suggesting that 57% of initiatives make their data open to the public in some form (Davis et al. 2023). In specific fields of citizen science, adherence to open data principles has been found to be even higher, with 69% of biodiversity citizen initiatives making data accessible (Suter et al. 2023). More than half of the marine citizen science initiatives are providing some degree of metadata that can be considered sufficient for making data findable. However, most of the initiatives do not provide a permanent data identifier, despite this being one of the requirements of Go-FAIR. This situation is not exceptional in citizen science, and, moreover, common for marine in situ data: they are traditionally collected by many different entities, resulting in data scattered throughout unconnected databases and repositories, often using incompatible formats, rendering the sharing of the information and data aggregation particularly challenging (Martín Míguez et al. 2019). Providing a semantic description of the observed properties is done only for 15.7% of the non-closed datasets, and, moreover, only six of the marine citizen initiatives in the inventory reference standard vocabularies. With such a low percentage, we can anticipate difficulties with integrating data from similar initiatives and in Digital Twins of the Ocean, as it is probable that they measure concepts that are incompatible or overlap only partially. Another difficulty with integrating the data collected by marine citizen scientists (or any other form of reuse) is the lack of reusability instructions in the form of a clear licence for 86.2% of the open or partially open initiatives. This limits the usefulness and the scope of the data gathered to the marine citizen science initiatives that capture them, making the initiatives the only actors capable of generating information and knowledge from the data. These findings confirm the argument by Bye et al. (2023) that marine citizen science initiative as a potential data source to support Digital Twins of the Ocean is a potential yet to be fully realised.

While there is room for improvement, this inventory shows that some projects have good data management procedures and follow the FAIR principles making their datasets mature enough to be present in international networks. For example, the European Marine Observation and Data Network (EMODnet) that includes a reference to 22 citizen science and participatory datasets in its catalogue^2^ and a handful of marine citizen science biodiversity datasets, can be found in the Global Biodiversity Information Facility (GBIF).

The discussion thus far highlights that metadata in the marine citizen science inventory (which is of interest to various actors, including researchers, authorities and citizens) are often not available without directly contacting the specific marine initiative(s). Several prior reviews of marine citizen science directly contacted initiative coordinators to gather additional information about initiatives (Theobald et al. 2015; Kelly et al. 2020; van Hee et al. 2020; de Grunt 2021; Garcia-Soto et al. 2021). In contrast, this review only includes information about each initiative that was available online in various forms (inventories, publications, conferences, etc.). As such, it provides a more accurate reflection of how easily researchers and other interested actors would be able to discover information about each initiative. However, this approach limited the level of detail that could be analysed. Initially, this review aimed to include details on the initiatives’ impact domains, participant dynamics, co-design process (if applicable), and context and setting. However, after an initial search, these fields were not included in the review because information was not easily discoverable for most initiatives. Considerable efforts were required over a lengthy period of time to generate even the basic information about each marine citizen science initiative included in the inventory.

The discussion, altogether, highlights that this cross-disciplinary study of the emergence and maturation of marine citizen science builds on, cuts across and advances aspects of marine science (e.g. the categorisation of marine citizen science initiatives according to specific marine disciplines and their geographic location), data science (e.g. FAIR and open marine citizen science data sets) and the science of citizen science (e.g. duration of marine citizen science initiatives, types of coordinators).

There are a number of limitations pertaining to the research presented here. Given the approach taken to identify marine citizen science initiatives either from the literature, their web presence or via other dissemination efforts (conferences, social media), the results may suffer from a bias towards larger, well-established or more ‘formalised’ initiatives since other initiatives may be less likely to have an online presence or an established communication strategy. Indeed, for 75 initiatives, this study could find no detailed information online, although their existence was ascertained via one of the above data collection approaches. Furthermore, the results presented here suffer from a bias towards English speaking initiatives, although an attempt was made to correct this by searching national repositories of marine citizen science initiatives, and by searching non-English review papers.

## Conclusions

Marine citizen science initiatives around the globe are on the increase, many are sustained over long periods of time, and they are of increasing relevance for marine science; yet the uptake of marine citizen science data is hampered by their ‘closedness’ and poor data management practices—they are far from being fully open and following the FAIR principles—as well as by difficulties with their appropriate categorisation, affecting all future uses (both intended and unplanned).

More effort needs to be made in making the data from marine citizen science initiatives FAIR. In particular, initiatives should define the observed properties based on standard vocabularies (e.g. link to concepts in the Marine Metadata Interoperability Ontology Registry and Repository,^3^ and open licences (such as Creative Commons)^4^ that facilitate reuse should be decided at the beginning of the initiatives or campaigns.

The structure for marine citizen science metadata inventory proposed and used by this study, together with the content of the information about 1267 marine citizen science initiatives collected, provides the basis for an interdisciplinary, dynamic online database (i.e. the MARCSI) that can be enhanced cumulatively by adding new initiatives. Such additions can be done by comprehensive follow-up studies or by the marine citizen science initiative initiators themselves, if technical means are made available and the criteria for determining the different levels within each category of classification are followed. Indeed, the coordinators and/or participants of already identified marine citizen science initiatives could provide further details to their initiative’s entries. This would help make marine citizen science initiatives findable, while also connecting and building the marine citizen science community globally as well as locally.

We are only at the start of understanding how marine citizen science changes the production of knowledge in marine science. As in other areas of citizen science, the process by which such initiatives are started, implemented, maintained over time and managed to link to science and policy processes, matters (Wehn 2022). In order to accelerate the increase of marine citizen science initiatives, especially by contributing to much needed knowledge about the ocean, further research on their dynamics is required. Knowing which marine citizen initiatives have existed and which are continuing, is an essential starting point; this is what the MARCSI provides.

## Supplementary Information

Below is the link to the electronic supplementary material.

Supplementary file1 (PDF 679 KB)

## Data Availability

The data set created, i.e. the inventory of marine citizen science initiatives, is available on Zenodo: Wehn, U., Bilbao, A., Somerwill, L., Linders, T., Maso, J., Parkinson, S, Semasingha, S., Woods, S. (2024) MARCSI—Inventory of Marine Citizen Science Initiatives, https://zenodo.org/records/14260016.

## Footnotes

1 wavelinks inventory.

2 https://emodnet.ec.europa.eu/geonetwork.

3 https://mmisw.org/.

4 https://creativecommons.org/licenses/.
