# Supplementary material for: Past and present marine citizen science around the globe: A cumulative inventory of initiatives and data produced
Source: Ambio. 2025 Feb 3;54(6):994–1009. doi: 10.1007/s13280-024-02119-z (PMC12055671; doi:10.1007/s13280-024-02119-z)
Supplement: Supplementary file 1 — Supplementary file1 (PDF 679 KB) [file 13280_2024_2119_MOESM1_ESM.pdf]

Ambio

Supplementary Information *This supplementary information has not been peer reviewed.*

Title **Past and present marine citizen science around the globe: a cumulative inventory of initiatives and data produced**

Table S1

General information and the data sections of the marine citizen science inventory (MARCSI)

**Field name**	**Field description**
Marine citizen science initiative title	Short version of title of the initiative as used in its own communication
Description / initiative summary	1 or 2 sentences describing the project/initiative
Initiative topic / keywords	Many topics collected and other keywords defining the project
Scientific topic	One of the following values: "Archaeology", "Biodiversity", "Ecology", "Environmental variables", "Fisheries", "Pollution", or "Single species".<br>Based on the structure followed on Garcia-Soto et al. 2021 or Van hall larensten (2020)<br>(Multiplicity: 3)
Start Date	Date when the project/initiative started
End date	Date when the project/initiative ended or the word “present” if it is still ongoing
Aim / intention / purpose of citizen science initiative / project	Aim, Intention or purpose of citizen science initiative/project
Marine focus area	One of the following values: "On shore", "Near shore", "Off shore", "On shore & Near shore", "On shore & Off shore", "Near shore & Off shore", or "On shore & Near shore & Off shore"
Geographical scale	Geographical scale of data collection. One of the following values:<br>"Global": Focus area can be/includes anywhere in the world<br>"International": Focus area is in multiple, specific countries (e.g. the Netherlands, Belgium, France)<br>"National": Focus area is spread across one country (e.g. the Netherlands)<br>"Regional": Focus area is spread across a large area within one country (e.g. a US state)<br>"Local": Focus area is specific to a small location within one country (e.g. a city or beach)
Geographic location - country/countries	Location of the data collection. Specific countries name or global category if it applies to several countries
Initiative documentation	Link to documentations about the project/initiative (e.g.

	website, deliverables etc)
Initiative host / coordinator	Name of the host or coordinator organisation
Initiative contact and email	Name of a contact person, organization or email
Sponsor / funding	Information about the source of funding of the initiative
Status	One of the following values: "abandoned", "active", "completed", "on hold", or "periodically active"

Data	
Data collection methodology	Methodology used to collect the data (e.g. a mobile app, chemical samples, etc)
Type of data collected	Variables and parameters in the data collected
Quantity of data collected	Number of data records collected
Indicators used	Derived information parameters from the data collected
Open access	One of the following values: “Yes, raw data is available”, “Partially (e.g. some data available in a report/paper etc)”, or “No”
Findable	"Yes" if comprehensive metadata and a persistent identifier was found (Provided only if the data is open access)
Accessible	"Yes" if the data was accessible by URL or web service. (Provided only if the data is open access)
Interoperable	"Yes" if a link to standard vocabularies to define the observed properties was found. (Provided only if the data is open access)
Reusable	"Yes" if a reference to a licence for the data was found. (Provided only if the data is open access)
Distribution	Link to a shared resource
Licence	Licence used to share the data
